# Achieving Better Function through Combining Orthodontics and Restorative Dentistry in the Case of Dental Abrasions

**DOI:** 10.1155/2019/8137585

**Published:** 2019-03-05

**Authors:** Laís C. Giacobbo, Lara Karolina Guimarães, Isabelle Adad Fornazari, Eduardo Monteiro Meda, Orlando Motohiro Tanaka

**Affiliations:** Pontifícia Universidade Católica do Paraná, School of Life Sciences, Curitiba, Brazil

## Abstract

This case report describes the orthodontic treatment used to prepare for restorative dentistry, aimed at the functional rehabilitation of an adult patient with Class I malocclusion, right posterior crossbite, and significant tooth wear on the palatal face of the maxillary canine to canine. The orthodontic treatment was performed with a total fixed appliance mini-expandex-type expander, associated with vertical elastics. Good dental intercuspation was obtained and enabled conditions for the restoration of the abrasions on the palatal surfaces of the maxillary incisors and canines and the tips of the buccal cusps of the maxillary right premolars and first molar. Excellent results were found during a 37-month follow-up.

## 1. Introduction

Gradual attrition of the occlusal surfaces of the teeth appears to be a general physiological phenomenon in all mammals, in every civilization, and at all ages [1]. Tooth wear in malocclusion patients should not be considered pathological but rather a consequence of the different interocclusal tooth arrangement [1].

Jang et al. reported that quantitative analysis of tooth wear is a function of orthodontic treatment modality [2]. Other studies have described tooth wear caused by orthodontic treatment, which should be of concern to orthodontists [3]. Apart from these, there are relatively few studies of tooth wear in the literature.

The aim of this article is to describe the orthodontic treatment, and functional rehabilitation, of an adult patient, skeletal and dental Class I, with posterior crossbite and significant abrasion on the palatine face of the maxillary canines, incisors, and right premolars and molars.

## 2. Case Report

A 38-year-old man came for orthodontic treatment with tooth abrasion as his chief complaint. His medical and dental histories showed good general health, except for a stressful lifestyle. He did not report any mandibular movements with nocturnal bruxism but did report functional movements or clenching in the daytime. He also stated that, when brushing, he applies too much force.

**Figure 1:**
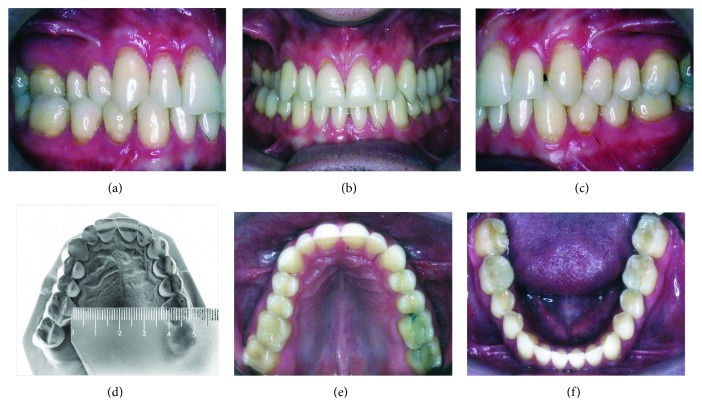
Pretreatment intraoral and maxillary dental cast photographs.

**Figure 2:**
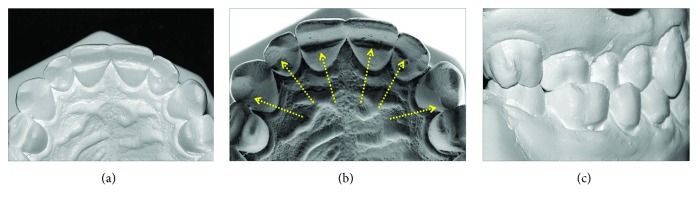
Pretreatment dental casts showing abrasions and posterior crossbite.

Dental analysis showed an Angle Class I malocclusion, normal overjet, a moderate overbite, and a slightly maxillary midline, shifted to the right (a posterior crossbite on the right side involved the first molar and both bicuspids (Figure 1(a)). Gingival retraction and dental abrasion in the facial cervical region were localized in the cervical areas of the maxillary canines and incisors (Figure 1(b)). There was wear on the occlusal and tip of the canines, premolars, and molars (Figures 1(a), 1(e), 1(f), 2(a), and 2(b)) and severely worn facets on the palatal surface of the maxillary canines and incisors, due to probable strong contact with the tip of the mandibular incisors (Figure 2(b)).

**Figure 3:**
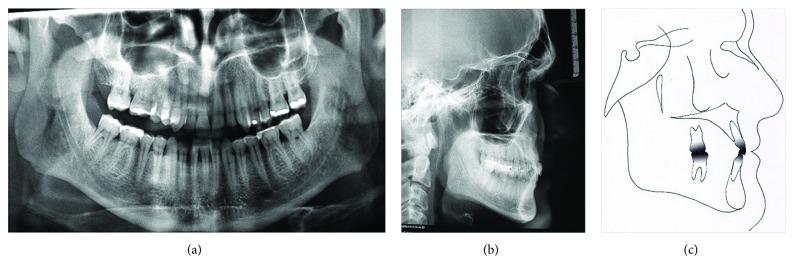
Pretreatment cephalometric and panoramic radiograph.

During the clinical examination, the patient had excellent plaque control and good gingival health. The cephalometric analysis showed a skeletal Class III relationship (ANB = -1°; AoBo = -5 mm), brachyfacial pattern (FMA = 17°), upright mandibular incisors (1-NB = 15°), and a concave profile (*Z*-angle = 78°) (Table 1). The panoramic radiograph showed extensive restorations on the maxillary and mandibular molars (Figure 3(a)).

The primary goals of the treatment proposed were as follows: transverse skeletal expansion of the maxilla, creation of a condition to restore the palatal side of the maxillary canine and incisors, and transverse dentoalveolar expansion of the maxillary right side.

Based on the treatment objectives, the following treatment options were suggested: surgically assisted maxillary expansion, associated with a hyrax-type expander; transpalatal arch; intermaxillary elastics; expansion with arches and intermaxillary elastics; or mini-expandex-type expander for dentoalveolar expansion.

**Figure 4:**
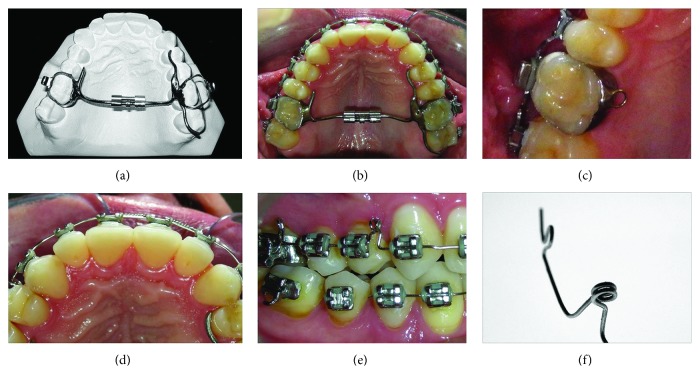
Progress details. Restoration of the palatal surface.

The patient did not accept the surgically assisted palatal expansion, so the first step was to affix the fixed mini-expandex-type expander to the maxillary first molars. On the left side, an extension was established on the second premolar and the second molar to increase the anchorage (Figures 4(a) and 4(b)). The activation was once daily for 10 days. After one month, fixed 0.022 in × 0.028 in standard edgewise brackets were bonded first on the maxillary arch, and 45 days later, on the mandibular arch. A 0.016 in SS archwire, with a mesially bent helicoidal loop, was used to jump both premolars, buccally (Figures 4(e) and 4(f)). After removing the mini-expandex, vertical crossbite elastics were attached to the archwire (Figure 4(c)).

**Figure 5:**
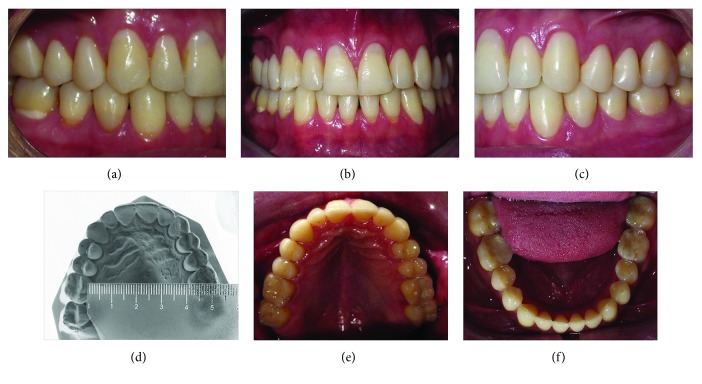
Posttreatment intraoral and maxillary dental cast photographs.

After completing the orthodontic treatment, three sessions of dental whitening were performed, with 38% hydrogen peroxide. The technique of direct restoration of the composite resin in the anterior (palatal surface) and posterior (cusp tip and cervical/facial lesion) teeth, using a universal adhesive system, allowed a better control of the restorative sequence [4]. The restorations were performed to the enamel and dentin and then photopolymerized for 10 seconds. The resins used were Charisma (Heraeus/Kulzer) on color OA2, Amelogen (Ultradent) color A2, and Durafill (Heraeus/Kulzer) color A2. After 14 days, the finishing and polishing of the composite resin restorations were carried out, achieving an excellent aesthetic result with this restorative technique (Figure 5).

The posttreatment records showed the correction of the maxillary transverse deficiency and the restoration of palatal surface abrasion of the maxillary canine and incisors. Ideal overjet and overbite relationships were maintained, with good intercuspation. The buccal cuspids of the maxillary right molar and premolars were built-up with composite resins (Figure 5). Retention was performed by removal of the wraparound-type appliance on the maxillary arch and bonded lingual arch, from the mandibular canine to canine. The treatment time was nine months.

**Figure 6:**
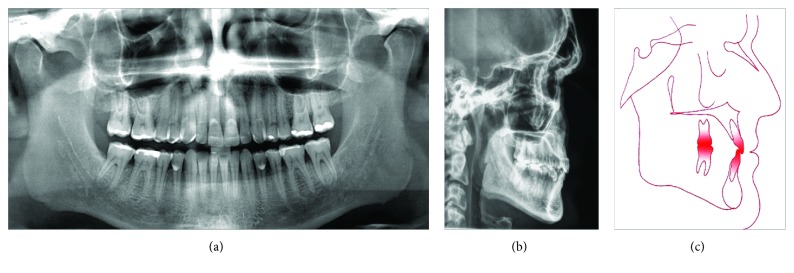
Posttreatment cephalometric and panoramic radiograph.

The cephalometric analysis did not show changes in the skeletal relationships and did show maintenance of the incisors' inclinations (Figures 6(b) and 6(c) and Table 1). The panoramic radiograph showed no significant root resorption (Figure 6(a)). The transverse dimension was changed slightly after treatment, where the intermolar width was slightly changed from 37.0 mm (Figure 1(d)) to 41.0 mm (Figure 5(d)).

**Figure 7:**
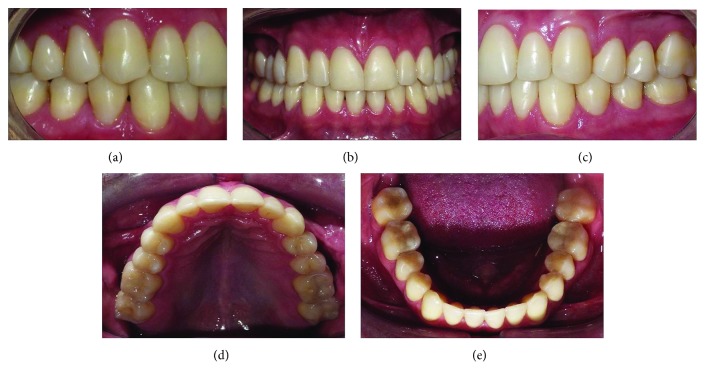
Follow-up intraoral photographs at 37 months.

After 37 months, follow-up photographs showed a stable occlusion, with maintenance of the overjet and overbite, as well as the molar and canine relationships, except for a slight rotation of the mandibular left central incisor. The patient stopped using the retainer one year after debonding (Figure 7).

## 3. Discussion

This clinical report demonstrates an approach to restoring severely worn maxillary anterior teeth with minimum dental preparation and adhesively bonded facial and palatal composite resins.

Tooth wear on malocclusion subjects should not be considered pathological but rather the consequence of different interocclusal arrangements [5]. In this case report, the patient was Class I malocclusion and showed good general health, except for a stressful lifestyle, clenching during the daytime, and a habit of applying too much force when brushing his teeth.

Successful dentoalveolar maxillary expansion with a mini-expandex-type palatal expander, associated with vertical elastics, was achieved, enabling restoration of the abraded teeth. The restorative management of the worn dentition involved working with a conformative treatment, involving restoration of the affected teeth without changing the occlusal vertical dimension and conforming to the intercuspal occlusal relationship that the patient presented [6].

Tooth wear can be described as the loss of hard dental tissue, resulting from physical or chemical attack; it is an all-embracing term, used to describe the combined processes of abrasion, erosion, and attrition [7].

Our patient presented with a strong brachyfacial pattern, and the tooth wear was evident on the crossbite side and into the palatal surface of the maxillary canines and incisors. This differs from the results of Kiliaridis et al., who found no significant differences in the sagittal relationships between persons with advanced wear and normal standards [8].

A comprehensive treatment plan was established, with coherent objectives and a clear understanding of the patient's expectations, including an assessment of the patient's ability to adapt to the treatment at the beginning, and involving a multidisciplinary approach to achieve and maintain a successful outcome.

## 4. Conclusion

After 37 months, follow-up photographs showed a stable occlusion, with maintenance of the overjet, overbite, restorations, and the molar and canine relationships

## Figures and Tables

**Table 1:** Cephalometric measurements.

Measurements	Norm	Initial	Final
SNA angle (°)	82°	85	84
SNB angle (°)	80°	86	85
ANB angle (°)	2°	-1	-1
Ao-Bo	♀ 0 ± 2 mm	-5	-6
	♂ 1 ± 2 mm		
Facial angle (°)	87°	93	91
Convexity (°)	0°	-5	-5
FMA (°)	25°	17	18
GoGn-SN (°)	32°	19	21
*Y*-axis (°)	59°	56	57
1-NA (mm)	22°	6	6
1-NA (°)	4 mm	23	21
1-NB (mm)	25°	2	3
1-NB (°)	4 mm	15	13
IMPA	90°	90	85
Interincisal angle (°)	132°	145	148
*Z*-angle (°)	75°	78	91
